# Association of decreased estimated glomerular filtration rate with lung cancer risk in the Korean population

**DOI:** 10.4178/epih.e2024041

**Published:** 2024-03-20

**Authors:** Soonsu Shin, Min-Ho Kim, Chang-Mo Oh, Hyejin Chun, Eunhee Ha, Hyo Choon Lee, Seong Ho Moon, Dong-Young Lee, Dosang Cho, Sangho Lee, Min Hyung Jung, Jae-Hong Ryoo

**Affiliations:** 1 Department of Preventive Medicine, Graduate School, Kyung Hee University, Seoul, Korea; 2 Department of Occupational and Environmental Medicine, Kyung Hee University Hospital, Seoul, Korea; 3 Ewha Medical Data Organization, Ewha Womans University Seoul Hospital, Seoul, Korea; 4 Department of Preventive Medicine, Kyung Hee University School of Medicine, Seoul, Korea; 5 Department of Family Medicine, Ewha Womans University College of Medicine, Seoul, Korea; 6 Department of Occupational and Environment Medicine, Ewha Womans University College of Medicine, Seoul, Korea; 7 Department of Internal Medicine, Veterans Healthcare Service Medical Center, Seoul, Korea; 8 Department of Neurosurgery, Ewha Womans University Seoul Hospital, Seoul, Korea; 9 Department of Anesthesiology and Pain Medicine, Kyung Hee University Medical Center, Seoul, Korea; 10 Department of Obstetrics and Gynecology, Kyung Hee University Hospital, Seoul, Korea; 11 Department of Occupational and Environmental Medicine, Kyung Hee University School of Medicine, Seoul, Korea

**Keywords:** Neoplasms, Cohort studies, Glomerular filtration rate, Lung neoplasms

## Abstract

**OBJECTIVES:**

Inconsistent results are available regarding the association between low estimated glomerular filtration rate (eGFR) and lung cancer risk. We aimed to explore the risk of lung cancer according to eGFR category in the Korean population.

**METHODS:**

We included 358,293 adults who underwent health checkups between 2009 and 2010, utilizing data from the National Health Insurance Service-National Sample Cohort. Participants were categorized into 3 groups based on their baseline eGFR, as determined using the Chronic Kidney Disease Epidemiology Collaboration equation: group 1 (eGFR ≥90 mL/min/1.73 m^2^), group 2 (eGFR ≥60 to <90 mL/min/1.73 m^2^), and group 3 (eGFR <60 mL/min/1.73 m^2^). Incidences of lung cancer were identified using the corresponding codes from the International Classification of Diseases, 10th revision. Multivariate Cox proportional hazard models were employed to calculate the adjusted hazard ratios (HRs) and 95% confidence intervals (CIs) for lung cancer incidence up to 2019.

**RESULTS:**

In multivariate analysis, group 2 exhibited a 26% higher risk of developing lung cancer than group 1 (HR, 1.26; 95% CI, 1.19 to 1.35). Furthermore, group 3 demonstrated a 72% elevated risk of lung cancer relative to group 1 (HR, 1.72; 95% CI, 1.58 to 1.89). Among participants with dipstick proteinuria of 2+ or greater, group 3 faced a significantly higher risk of lung cancer than group 1 (HR, 2.93; 95% CI, 1.37 to 6.24).

**CONCLUSIONS:**

Low eGFR was significantly associated with increased lung cancer risk within the Korean population. A particularly robust association was observed in individuals with severe proteinuria, emphasizing the need for further investigation.

## GRAPHICAL ABSTRACT

[Fig f1-epih-46-e2024041]

## Key Message

Our research found that lower estimated glomerular filtration rate with proteinuria increased the risk of lung cancer in a Korean population. These findings suggest that decreased kidney function may increase the risk of lung cancer, indicating the need for careful observation of patients with impaired kidney function.

## INTRODUCTION

Lung cancer represents one of the most common cancers and the leading cause of cancer-related deaths globally, accounting for an estimated 18.0% of all such deaths in 2020 [1]. In recent years, considerable effort has been dedicated to the prevention and early detection of lung cancer through initiatives such as smoking cessation programs and screening with low-dose computed tomography. Consequently, the incidence and mortality rates of lung cancer have been declining among men in high-income countries [2,3]. However, a different trend is evident for women in most nations, with both incidence and mortality rates on the rise [3]. Furthermore, a growing proportion of patients diagnosed with lung cancer have never smoked [4]. This trend underscores the need to identify additional risk factors for lung cancer beyond tobacco use.

Chronic kidney disease (CKD) is diagnosed based on a decline in the estimated glomerular filtration rate (eGFR) or an increase in albuminuria over a 3-month period [5]. Retrospective cohort studies have indicated an increased risk of both overall cancer incidence and mortality among individuals with decreased eGFR [6-12]. However, other studies have reported conflicting results [13-17]. While some research has indicated a potential link between impaired kidney function and an elevated risk of lung cancer [6,7,9,10,18], these associations have not been consistently observed across studies [8,12,14,16,17]. Previous investigations have explored the relationship between impaired kidney function and various types of cancer concurrently, precluding adjustment for lung cancer-specific covariates. Additionally, the issue of multiple comparisons may have contributed to the inconsistency across findings. A number of studies did not incorporate a competing risk analysis [8,10,14,16,18], which is noteworthy given that the mean age at lung cancer diagnosis in the United States is 70 years [19]. Thus, previous studies may conceivably have overestimated the risk of lung cancer [20].

In this study, we investigated the association between low eGFR and lung cancer risk using a multivariate Cox proportional hazards model within the Korean population. We conducted a competing risk analysis to account for deaths that occurred during the follow-up period. Furthermore, we evaluated the risk of lung cancer according to eGFR category after stratification by urine protein level. Proteinuria serves as an indicator of kidney health and can signal a decline in kidney function prior to a decrease in eGFR [21,22]. Through these stratified analyses, we aimed to provide evidence regarding the association between impaired kidney function and increased risk of lung cancer. The findings may be useful in assessing lung cancer risk in patients who have been incidentally identified with decreased kidney function.

## MATERIALS AND METHODS

### Database

In this retrospective cohort study, we utilized data from the National Health Insurance Service National Sample Cohort (NHIS-NSC) from 2002 to 2019. This information is maintained by the National Health Insurance Service (NHIS) in Korea. The NHIS-NSC represented approximately 2.2% of the Korean population in 2002, with follow-up conducted through 2019 [23]. The NHIS provides a free biennial health checkup for insured individuals who are at least 40 years of age. It is also tasked with the collection and documentation of health checkup results, as well as information pertaining to the utilization of medical services, including diagnoses and treatments. In recent years, the NHIS has provided a sample cohort database for research purposes, from which personal identification details have been omitted. The NHIS-NSC database includes data on health checkups and medical service utilization, such as records of hospital visits, diagnoses, and treatments [23].

### Study population

For the analysis, we included a cohort of 362,285 participants who underwent health checkups between 2009 and 2010 (Supplementary Material 1). We excluded 3,877 individuals with a history of lung cancer prior to the health checkups, as identified within the NHIS-NSC claims data by the International Classification of Diseases, 10th revision, Clinical Modification (ICD-10) code C34 (malignant neoplasms of the bronchi and lungs) [24]. Additionally, we excluded 115 participants due to missing serum creatinine (SCr) levels. Our final analysis comprised 358,293 participants. We monitored the incidence of lung cancer using the ICD-10 codes recorded in the database. The total follow-up period amounted to 3,470,426.78 person-years, with an average follow-up duration of 9.69 years (standard deviation [SD], 1.55).

### Exposure

Kidney function is assessed using eGFR, which can be calculated using the Chronic Kidney Disease Epidemiology Collaboration (CKD-EPI) equation [25]. According to the Kidney Disease: Improving Global Outcomes (KDIGO) clinical practice guidelines, GFR is categorized as follows: G1 (normal or high, ≥ 90 mL/min/ 1.73 m^2^), G2 (mildly decreased, 60-89 mL/min/1.73 m^2^), G3a (mildly to moderately decreased, 45-59 mL/min/1.73 m^2^), G3b (moderately to severely decreased, 30-44 mL/min/1.73 m^2^), G4 (severely decreased, 15-29 mL/min/1.73 m^2^), and G5 (kidney failure, < 15 mL/min/1.73 m^2^) [26]. The KDIGO guidelines recommend that individuals with an incidentally observed GFR below 60 mL/min/1.73 m^2^ (encompassing GFR categories G3a to G5) should undergo ongoing renal function monitoring [26]. Consequently, study participants were categorized based on their eGFR levels (≥ 90, ≥ 60 to < 90, or < 60 mL/min/1.73 m^2^) as recorded during the health checkups in 2009-2010.

### Covariates

Covariates were identified using a directed acyclic graph and included sex, body mass index (BMI), fasting blood glucose level, gamma-glutamyltransferase (GGT) level, smoking amount (measured in pack-years), smoking status, alcohol consumption, engagement in physical activity, chronic obstructive pulmonary disease (COPD), and asthma (Supplementary Material 2).

At the health visits, participants first completed a questionnaire covering their medical history and lifestyle habits. A doctor then reviewed the responses during the consultation to ensure accuracy. The questionnaire inquired about smoking history, alcohol consumption, and physical activity level. Smoking amount, quantified in pack-years, was determined using a specific smoking-related questionnaire. Smoking status was classified into 3 categories. According to the World Health Organization definitions, never-smokers are individuals who have smoked fewer than 100 cigarettes in their lifetime [27]. Former smokers are those who have quit smoking, while current smokers are those who continue to smoke. Alcohol consumption was characterized as drinking alcohol more than twice per week. Physical activity was defined as participating in moderate-intensity exercise for at least 30 minutes per day on 5 or more days per week.

Anthropometric measurements and laboratory tests were also conducted. Trained examiners measured participants’ weight and height to calculate BMI by dividing weight (in kilograms) by the square of height (in meters). Additionally, systolic and diastolic blood pressure were measured by these examiners. During the health checkup, various laboratory tests were performed, including assessments of fasting blood glucose, total cholesterol, triglycerides, high-density lipoprotein cholesterol, low-density lipoprotein cholesterol, SCr, aspartate aminotransferase, alanine aminotransferase, and GGT levels.

In our multivariate analyses, we adjusted for potential confounding factors associated with lung cancer, including COPD and asthma. These chronic conditions are characterized by persistent lung inflammation and are known to increase the risk of lung cancer [28,29]. Within the sample cohort database, participant diagnoses were determined using ICD-10 codes. COPD was identified by the ICD-10 codes J40 through J44 [24], while asthma was determined by the code J45 [24]. Participants were considered to have COPD or asthma if they had at least 1 inpatient or outpatient visit with the corresponding ICD-10 code(s) prior to the baseline.

### Outcome

The primary outcome of the study was the incidence of newly diagnosed lung cancer. Lung cancer was identified using the ICD10 code C34—indicating malignant neoplasm of the bronchus and lung [24]—as registered in the NHIS-NSC claim data. Within the NHIS data, the operational definition of lung cancer, based on the ICD-10 code for the primary diagnosis, demonstrated a sensitivity of 95.0% (95% confidence interval [CI], 94.8 to 95.1) and a positive predictive value of 88.9% (95% CI, 88.8 to 89.1) [30]. Participants were monitored for the incidence of lung cancer from their health visits in 2009-2010 until December 31, 2019.

### Statistical analysis

The baseline characteristics were summarized using means±SDs for continuous variables and percentages for categorical variables. Differences in baseline characteristics were assessed with one-way analysis of variance and the chi-square test. Person-years were calculated as the total follow-up time until the incidence of lung cancer, loss to follow-up, death, or December 31, 2019.

The cumulative incidence of lung cancer across eGFR groups was compared using Kaplan–Meier plots and log-rank tests. Cox proportional hazard models were employed to evaluate the association between eGFR group and lung cancer incidence. Adjusted hazard ratios (HRs) and 95% CIs were computed using a multivariate model that accounted for confounding factors. As a key component of the CKD-EPI equation, age was not incorporated into the multivariate model.

Our analysis was stratified based on urine protein levels, with participants divided into 2 groups: those with a urine protein level of 2+ or higher and those with a level below 2+. Urine protein levels were assessed using a dipstick test, which yields results on a graded scale with options of absent, trace, 1+, 2+, 3+, and 4+.

Within our sensitivity analysis, we carried out a competing risk analysis using Fine-Gray subdistribution hazard models. This was done to assess the risk of incident lung cancer while accounting for death as a competing event during the follow-up period [31]. To minimize the likelihood of reverse causality affecting our results, we conducted further analyses. These analyses involved excluding participants who received a lung cancer diagnosis within the first year of follow-up—resulting in the exclusion of 531 participants—and within the first 5 years of follow-up, which led to the exclusion of 3,135 participants. A p-value of less than 0.05 was considered to indicate statistical significance, and all analyses were performed using SAS version 9.4 (SAS Institute Inc., Cary, NC, USA).

### Ethics statement

Ethical approvals for the data collection and analyses were received from the Institutional Review Board (IRB) of Kyung Hee University Hospital. Due to the retrospective nature of the study and its use of a de-identified database, the requirement for informed consent was waived by the IRB (IRB No. KHUH 2023-04-023).

## RESULTS

Table 1 presents the baseline characteristics of the study participants, stratified by eGFR group. Over a follow-up period of 3,470,426.78 person-years, we identified 5,757 new cases of lung cancer. The mean± SD participant age and BMI at baseline were 58.77± 8.94 years and 24.01± 2.92 kg/m^2^, respectively. The distribution of participants among the eGFR groups was as follows: group 1 (eGFR ≥90 mL/min/1.73 m^2^) included 114,986 participants, group 2 (eGFR ≥ 60 to < 90 mL/min/1.73 m^2^) comprised 208,708 participants, and group 3 (eGFR < 60 mL/min/1.73 m^2^) contained 34,599 participants. All variables in Table 1 demonstrated statistically significant differences across the 3 eGFR groups. Compared to group 1, the lower eGFR groups had an older average age and a smaller proportion of individuals who currently smoked or consumed alcohol. Additionally, higher rates of COPD and asthma were observed in the lower eGFR groups.

The Kaplan-Meier curves illustrate the cumulative incidence of lung cancer by eGFR group (Supplementary Material 3). Compared to group 1, the log-rank test results revealed a significantly higher risk of lung cancer in the lower eGFR groups (p< 0.001) (Supplementary Material 3). Table 2 presents the HRs and 95% CIs for incident lung cancer for each of the 3 baseline eGFR groups. In the unadjusted model, relative to group 1, the HRs for lung cancer incidence were 1.28 (95% CI, 1.20 to 1.36) for group 2 and 1.75 (95% CI, 1.61 to 1.91) for group 3 (p for trend < 0.001). In the multivariate model, the adjusted HRs for lung cancer incidence were 1.26 (95% CI, 1.19 to 1.35) for group 2 and 1.72 (95% CI, 1.58 to 1.89) for group 3, again in reference to group 1 (p for trend < 0.001). Supplementary analyses excluding participants who developed lung cancer within 1 year (Supplementary Material 4) and 5 years (Supplementary Material 5) after entering the cohort showed an increased adjusted HR in the groups with lower eGFR. The results of the competing risk analysis aligned with the main analysis (Supplementary Material 6), indicating that the adjusted HRs for incident lung cancer were significantly higher in group 2 (1.23; 95% CI, 1.16 to 1.31) and group 3 (1.58; 95% CI, 1.44 to 1.73) relative to the highest eGFR group. In the sex-stratified analysis, the adjusted HRs for lung cancer were consistently higher in group 3 than in group 1 for both male and female participants (Supplementary Material 7).

In the analysis stratified by urine protein level, we observed similar associations between eGFR and the risk of lung cancer (Table 3). Among participants with dipstick proteinuria of less than 2+, those in the lower eGFR groups exhibited a higher risk of lung cancer compared to group 1. The adjusted HRs were 1.26 (95% CI, 1.18 to 1.34) for group 2 and 1.70 (95% CI, 1.56 to 1.87) for group 3 (p for trend < 0.001). Additionally, among participants with a urine protein level of 2+ or higher, the risk of lung cancer was greater in the lower eGFR groups than in group 1, according to the multivariate-adjusted model. The adjusted HRs for these groups were 2.00 (95% CI, 0.97 to 4.15) for group 2 and 2.93 (95% CI, 1.37 to 6.24) for group 3 (p for trend < 0.001).

## DISCUSSION

In this retrospective analysis of a cohort representative of the Korean population, we observed a significant association between reduced eGFR and an increased risk of lung cancer. Following sensitivity analyses aimed at minimizing reverse causality and competing risk bias, the relationship between low eGFR and lung cancer risk persisted as statistically significant.

The urine dipstick test is a straightforward and cost-effective method for detecting proteinuria in the general population. A urine dipstick result of 1+ is equivalent to approximately 30 mg/dL of protein, while 2+ corresponds to 100 mg/dL, 3+ to 300 mg/dL, and 4+ to 1,000 mg/dL [32]. Proteinuria, the abnormal loss of plasma proteins in the urine, can result from increased glomerular permeability or incomplete tubular reabsorption. The presence of proteinuria may be predictive of a rapid decline in kidney function [33]. Research has indicated that the general population with dipstick proteinuria of at least 1+ experience a more rapid decline in eGFR than those without proteinuria [34]. Consequently, kidney function assessment should consider both eGFR and proteinuria. In line with this, our stratified analysis revealed a marked increase in lung cancer risk among those with low eGFR and dipstick proteinuria of 2+ or greater. These findings may provide additional support for an association between impaired kidney function and elevated lung cancer risk.

Previous observational studies investigating the relationship between reduced kidney function and the risk of lung cancer have yielded inconsistent results. Jørgensen et al. [10] were the first to report that an elevated albumin-to-creatinine ratio was associated with a higher risk of lung cancer in a cohort of 5,425 Norwegians without diabetes. In a prospective cohort study involving 3,654 participants aged over 49 years, a higher risk of lung cancer was found among those with an eGFR of less than 40 mL/min/1.73 m^2^ [11]. Similarly, a recent retrospective study from the UK Biobank demonstrated a greater risk of lung cancer in participants with an eGFR of less than 60 mL/min/1.73 m^2^ [9]. In contrast, a cohort study of the Korean population indicated an elevated risk of lung cancer in individuals with 1+ proteinuria, but the association between low eGFR and lung cancer risk was not statistically significant [7]. Other observational studies have reported no significant association between impaired kidney function and the risk of lung cancer [8,12-14,16,17]. The conflicting results may be attributable to demographic differences and variations in study settings. Among cohort studies in Western populations, a decrease in eGFR has been associated with a significantly increased risk of lung cancer in studies with longer observation periods (median follow-up: 9.1-11.3 years) [9-11], but this association was not found in a study with shorter follow-up durations (median follow-up: 5.0-5.3 years) [8,14]. Observational studies conducted in East Asian populations, including those from Korea, Japan, China, and Taiwan, have also reported inconsistent findings regarding the correlation between reduced eGFR and the risk of lung cancer [6,7,12,16-18]. However, consistent with the findings of this study, research evaluating kidney function decline through the presence of proteinuria or albuminuria has consistently indicated an increased risk of lung cancer in patients with these conditions [6,7,10].

In the present study, the association between lower eGFR and elevated risk of lung cancer remained robust, even after sensitivity analyses. While the exact biological mechanisms underlying this association are not fully understood, several potential explanations have been proposed. The combined effects of inflammation and oxidative stress are considered pivotal in the development of cancer [35]. These may be exacerbated by reduced renal clearance in patients with CKD, thereby increasing their cancer risk [35]. In a study of 180 individuals with CKD, a distinct increase was observed in inflammatory markers such as cyclooxygenase-2 and inducible nitric oxide synthase, along with a downregulation of pro-oxidant genes and antioxidants [36]. Additionally, the Wnt/β-catenin signaling pathway was found to be activated in these patients [36]. Evidence suggests that this pathway plays a role in tumorigenesis. The concurrent activation of the KRAS and Wnt/β-catenin pathways has been reported to meaningfully contribute to lung tumor formation [37]. The aromatic hydrocarbon receptor (AHR), a ligand-dependent transcription factor, is known for its role in lung cancer induced by cigarette smoking [38]. Carcinogens in cigarettes, such as dioxins and benzopyrene, exert a nongenotoxic effect by binding AHR in human bronchial epithelial cells [38]. AHR activation influences a wide array of biological processes, including xenobiotic metabolism, inflammation, cell proliferation, and cell adhesion [39]. In patients with CKD, precipitated uremic toxins may activate AHR. Dou et al. [40] reported that patients exhibited elevated levels of serum AHR-activating potential prior to dialysis, with a decrease following the procedure. The study also revealed significant increases in serum urea concentration, AHR-activating potential, and *Cyp1a1* mRNA expression in mice subjected to 5/6 nephrectomy compared to sham-operated controls. Therefore, the activation of Wnt/β-catenin, KRAS, and AHR pathways due to impaired kidney function may play a substantial role in the development of lung cancer.

The primary strength of this study lies in its use of a multivariate model that incorporates smoking status, smoking amount, COPD, and asthma. By considering these confounding factors, we aimed to gain a more thorough understanding of the association between low eGFR and lung cancer risk. Another strength is the performance of long-term observation within a cohort representative of the Korean population. The analysis stratified by dipstick proteinuria results indicated that patients with severe proteinuria and low eGFR should be closely monitored for the potential development of lung cancer. This study provides valuable insights into the connection between decreased eGFR and lung cancer risk in an East Asian population. Periodic follow-up for lung cancer may be advisable in patients with reduced kidney function identified during health screenings or incidentally.

Our study did have certain limitations. First, the sample size was restricted to participants with markedly decreased eGFR ( < 30 mL/min/1.73 m^2^). Patients with end-stage renal disease, who typically receive regular medical care, may not have been included as they might not attend routine health checkups. Second, kidney function was assessed using only a single eGFR measurement, without accounting for temporal fluctuations. This approach may not fully reflect the chronicity of kidney function impairment. Nevertheless, a previous large cohort study indicated that low eGFR was not a transient condition [41]. Specifically, in a follow-up test conducted after 30 days, 68% of the 54,576 individuals with an initial eGFR below 80 mL/min/1.73 m^2^ demonstrated either a similar or a further reduced eGFR [41]. Third, our analysis did not differentiate between histological types of lung cancer. In Korea, non-small cell lung cancer constitutes approximately 85% of all lung cancer cases, with adenocarcinoma being the most common histopathological subtype, representing 36.1% of cases [42].

Our study revealed a strong association between reduced eGFR and a heightened risk of lung cancer, even after adjusting for specific confounders such as COPD and asthma. We found consistent results regarding the incidence of lung cancer in individuals with dipstick proteinuria of 2+ or greater. These findings suggest that impaired kidney function may represent a potential risk factor for lung cancer. Further research is needed to explore lung cancer risk among patients with CKD who have experienced impaired kidney function for at least 3 months. Recognizing that patients with diminished kidney function face an elevated risk of lung cancer and closely monitoring for the onset of the disease may represent an effective strategy.

## Figures and Tables

**Figure f1-epih-46-e2024041:**
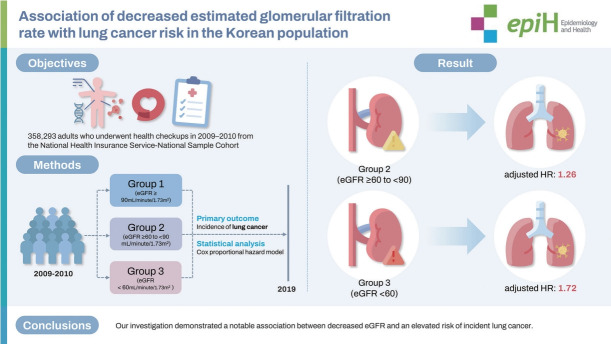


**Table 1.** Baseline characteristics of participants across the 3 baseline eGFR groups (n=358,293)

Characteristics	Overall	eGFR (mL/min/1.73 m^2^)			
		Group 1 (eGFR≥90, n=114,986)	Group 2 (60≤eGFR<90, n=208,708)	Group 3 (eGFR<60, n=34,599)	p-value^1^
Person-years (total)	3,470,426.78	1,127,480.18	2,021,331.03	321,615.57	
Person-years (average)	9.69±1.55	9.81±1.29	9.68±1.54	9.30±2.17	<0.001
Age (yr)	58.77±8.94	56.13±6.66	59.26±9.23	64.62±10.43	<0.001
Sex					<0.001
Male	194,063 (54.2)	58,894 (51.2)	117,031 (56.1)	18,138 (52.4)	
Female	164,230 (45.8)	56,092 (48.8)	91,677 (43.9)	16,461 (47.6)	
BMI (kg/m^2^)	24.01±2.92	23.86±2.93	24.06±2.90	24.24±3.03	<0.001
Systolic BP (mmHg)	125.29±15.27	124.38±15.05	125.39±15.26	127.65±15.76	<0.001
Diastolic BP (mmHg)	77.58±9.96	77.32±10.02	77.67±9.91	77.91±10.04	<0.001
Total cholesterol (mg/dL)	200.07±37.68	198.52±37.08	200.92±37.56	200.14±40.10	<0.001
Triglycerides (mg/dL)	117 (83-169)	113 (79-163)	118 (84-170)	127 (91-182)	<0.001
HDL cholesterol (mg/dL)	55.27±30.93	55.83±29.95	54.39±23.81	58.80±59.06	<0.001
LDL cholesterol (mg/dL)	118.62±38.16	117.31±38.84	119.38±37.75	118.41±38.25	<0.001
Fasting blood glucose (mg/dL)	100.99±25.25	100.21±25.18	100.79±24.44	104.81±29.57	<0.001
SCr (mg/dL)	1.09±1.33	0.73±0.13	0.96±0.15	3.07±3.71	<0.001
eGFR (mL/min/1.73 m^2^)	80.89±19.40	100.10±7.99	76.79±8.23	41.79±20.45	<0.001
Urine protein level					<0.001
Normal	338,290 (94.9)	109,735 (95.7)	197,817 (95.1)	30,738 (89.6)	
Trace	8,352 (2.3)	2,452 (2.1)	4,659 (2.2)	1,241 (3.6)	
1+	6,582 (1.8)	1,728 (1.5)	3,622 (1.7)	1,232 (3.6)	
≥2+	3,669 (1.0)	783 (0.7)	1,804 (0.9)	1,082 (3.2)	
AST (U/L)	24 (20-29)	23 (20-29)	24 (20-29)	24 (20-29)	<0.001
ALT (U/L)	21 (16-29)	21 (16-29)	21 (16-29)	20 (15-27)	<0.001
GGT (U/L)	24 (17-40)	24 (16-41)	25 (17-40)	24 (17-38)	<0.001
Smoking amount (pack-years)	7.52±13.83	7.37±13.62	7.65±13.87	7.16±14.26	<0.001
Smoking status					<0.001
Never smoker	227,475 (64.7)	73,908 (65.5)	130,661 (63.8)	22,906 (67.4)	
Former smoker	63,739 (18.1)	18,208 (16.1)	39,078 (19.1)	6,453 (19.0)	
Current smoker	60,346 (17.2)	20,656 (18.3)	35,088 (17.1)	4,602 (13.5)	
Alcohol consumption	49,034 (13.8)	17,230 (15.1)	28,250 (13.7)	3,554 (10.4)	<0.001
Physical activity	60,133 (17.0)	18,953 (16.7)	35,334 (17.2)	5,846 (17.1)	0.003
COPD	105,801 (29.5)	30,876 (26.8)	62,229 (29.8)	12,696 (36.7)	<0.001
Asthma	71,392 (19.9)	20,867 (18.1)	41,885 (20.1)	8,640 (25.0)	<0.001
Development of lung cancer	5,757 (1.6)	1,519 (1.3)	3,481 (1.7)	757 (2.2)	<0.001

Values are presented as the mean±standard deviation, number (%), or median (interquartile range). eGFR, estimated glomerular filtration rate; BMI, body mass index; BP, blood pressure; HDL, high-density lipoprotein; LDL, low-density lipoprotein; AST, aspartate aminotransferase; SCr, serum creatinine; ALT, alanine aminotransferase; GGT, gamma-glutamyltransferase; COPD, chronic obstructive pulmonary disease.

1 Using analysis of variance for continuous variables and the chi-square test for categorical variables.

**Table 2.** Hazard ratios and 95% confidence intervals for lung cancer incidence across the 3 baseline eGFR groups

Variables	Person-years	Incident cases	Incidence density (per 10,000 person-years)	Unadjusted model	Multivariate-adjusted model^1^
eGFR (mL/min/1.73 m^2^)					
Group 1 (≥90)	1,127,480.18	1,519	13.47	1.00 (reference)	1.00 (reference)
Group 2 (≥60 to <90)	2,021,331.03	3,481	17.22	1.28 (1.20, 1.36)	1.26 (1.19, 1.35)
Group 3 (<60)	321,615.57	757	23.54	1.75 (1.61, 1.91)	1.72 (1.58, 1.89)
p for trend				<0.001	<0.001
Sex (female vs. male)	-	-	-	-	0.56 (0.52, 0.61)
BMI	-	-	-	-	0.93 (0.92, 0.94)
Fasting blood glucose	-	-	-	-	1.00 (1.00, 1.00)
GGT	-	-	-	-	1.00 (1.00, 1.00)
Smoking amount (pack-years)	-	-	-	-	1.02 (1.02, 1.02)
Smoking status					
Never smoker	-	-	-	-	1.00 (reference)
Former smoker	-	-	-	-	0.75 (0.68, 0.82)
Current smoker	-	-	-	-	1.18 (1.08, 1.29)
Alcohol consumption	-	-	-	-	1.07 (0.99, 1.14)
Physical activity	-	-	-	-	1.02 (0.95, 1.09)
COPD	-	-	-	-	1.68 (1.59, 1.78)
Asthma	-	-	-	-	1.40 (1.32, 1.49)

eGFR, estimated glomerular filtration rate; BMI, body mass index; GGT, gamma-glutamyltransferase; COPD, chronic obstructive pulmonary disease.

1 The multivariate-adjusted model was adjusted for sex, BMI, fasting blood glucose, GGT, smoking amount (pack-years), smoking status, alcohol consumption, physical activity, COPD, and asthma; Age was not included in the multivariate-adjusted model.

**Table 3.** Hazard ratios and 95% confidence intervals for lung cancer incidence across the 3 baseline eGFR groups, after stratification by urine protein level

Variables	Urine protein level			
	<2+ (n=353,224, 98.97%)		≥2+ (n=3,669, 1.03%)	
	Unadjusted model	Multivariate-adjusted model^1^	Unadjusted model	Multivariate-adjusted model^1^
eGFR (mL/min/1.73 m^2^)				
Group 1 (≥90)	1.00 (reference)	1.00 (reference)	1.00 (reference)	1.00 (reference)
Group 2 (≥60 to <90)	1.27 (1.20, 1.35)	1.26 (1.18, 1.34)	1.95 (0.95, 4.03)	2.00 (0.97, 4.15)
Group 3 (<60)	1.73 (1.58, 1.89)	1.70 (1.56, 1.87)	2.94 (1.40, 6.17)	2.93 (1.37, 6.24)
p for trend	<0.001	<0.001	<0.001	<0.001
Sex (female vs. male)	-	0.56 (0.52, 0.61)	-	0.28 (0.13, 0.62)
BMI	-	0.93 (0.92, 0.94)	-	0.92 (0.85, 0.99)
Fasting blood glucose	-	1.00 (1.00, 1.00)	-	1.00 (1.00, 1.01)
GGT	-	1.00 (1.00, 1.00)	-	1.00 (0.99, 1.00)
Smoking amount (pack-years)	-	1.02 (1.02, 1.02)	-	1.00 (0.99, 1.02)
Smoking status				
Never smoker	-	1.00 (reference)	-	1.00 (reference)
Former smoker	-	0.74 (0.68, 0.82)	-	1.05 (0.51, 2.17)
Current smoker	-	1.18 (1.08, 1.28)	-	1.80 (0.89, 3.65)
Alcohol consumption	-	1.06 (0.99, 1.14)	-	1.47 (0.85, 2.52)
Physical activity	-	1.02 (0.95, 1.09)	-	1.09 (0.63, 1.91)
COPD	-	1.69 (1.60, 1.79)	-	1.19 (0.73, 1.96)
Asthma	-	1.40 (1.32, 1.50)	-	1.35 (0.78, 2.33)

eGFR, estimated glomerular filtration rate; BMI, body mass index; GGT, gamma-glutamyltransferase; COPD, chronic obstructive pulmonary disease.

1 The multivariate-adjusted model was adjusted for sex, BMI, fasting blood glucose, GGT, smoking amount (pack-years), smoking status, alcohol consumption, physical activity, COPD, and asthma; Age was not included in the multivariate-adjusted model.

## Supplementary materials

Supplementary Material 1. Diagram showing the process of selecting study participants.

Supplementary Material 2. Directed acyclic graph depicting the association between decreased eGFR, covariates and incident lung cancer.

Supplementary Material 3. Cumulative incidence of incident lung cancer by eGFR groups.

Supplementary Material 4. Hazard ratios and 95% confidence intervals for the incidence of lung cancer according to the three groups of baseline eGFR categories after excluding the possibility of 1-year reverse causality

Supplementary Material 5. Hazard ratios and 95% confidence intervals for the incidence of lung cancer according to the three groups of baseline eGFR categories after excluding the possibility of 5-year reverse causality

Supplementary Material 6. Hazard ratios and 95% confidence intervals for the incidence of lung cancer according to the three groups of baseline eGFR categories using competing risk analysis with death during follow-up

Supplementary Material 7. Hazard ratios and 95% confidence intervals for the incidence of lung cancer to the three groups of baseline eGFR categories after stratifying by sex
