# Supplementary material for: Association of decreased estimated glomerular filtration rate with lung cancer risk in the Korean population
Source: Epidemiol Health. 2024 Mar 20;46:e2024041. doi: 10.4178/epih.e2024041 (PMC11369561; doi:10.4178/epih.e2024041)
Supplement: Supplementary Material 1. — Diagram showing the process of selecting study participants. [file epih-46-e2024041-Supplementary-1.docx]

**
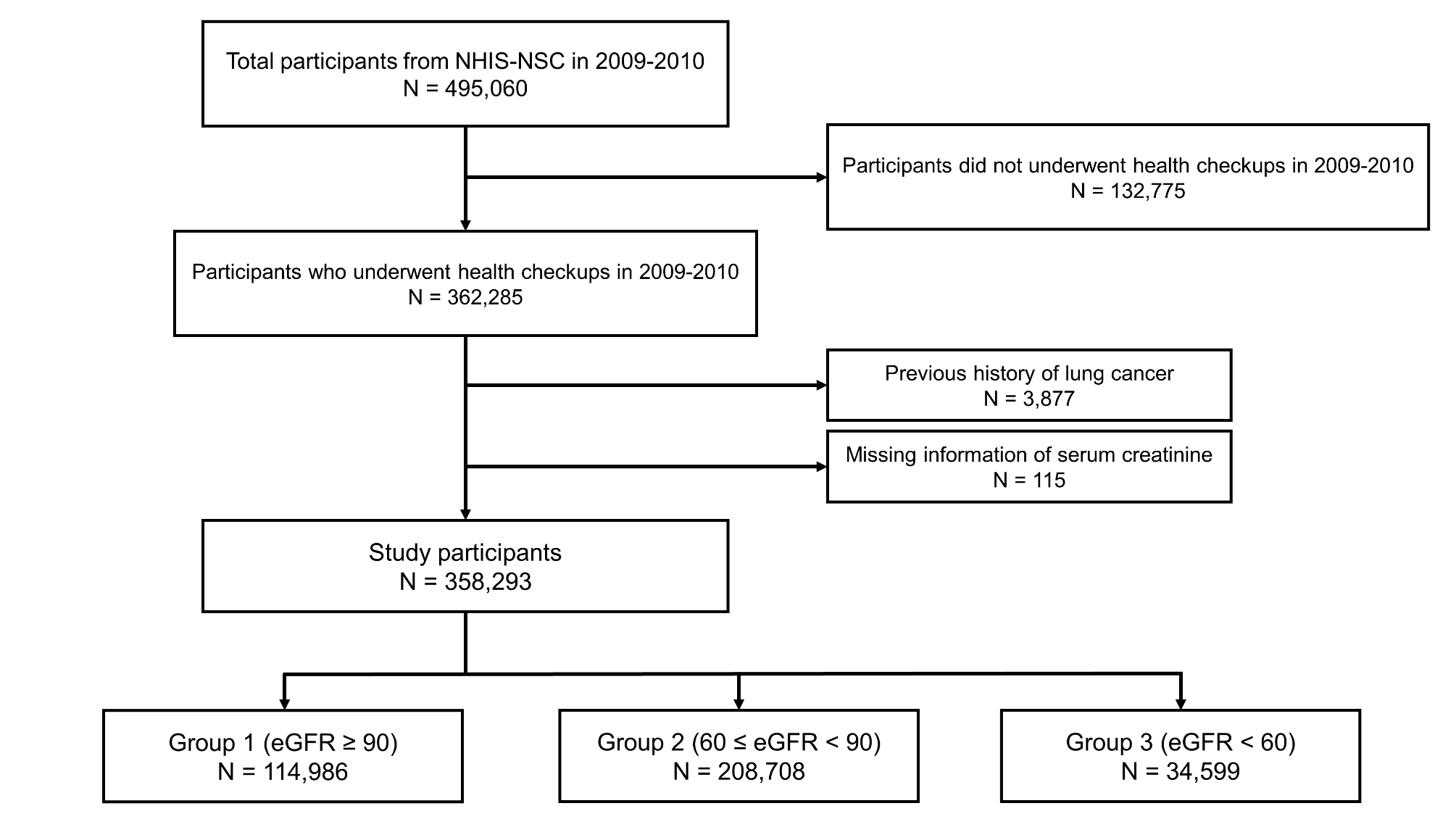
**

**Supplementary Material 1. Diagram showing the process of selecting study participants.**

Abbreviations: NHIS-NSC, National Health Insurance Service-National Sample Cohort; eGFR, estimated glomerular filtration rate
