# Supplementary material for: Association of decreased estimated glomerular filtration rate with lung cancer risk in the Korean population
Source: Epidemiol Health. 2024 Mar 20;46:e2024041. doi: 10.4178/epih.e2024041 (PMC11369561; doi:10.4178/epih.e2024041)
Supplement: Supplementary Material 2. — Directed acyclic graph depicting the association between decreased eGFR, covariates and incident lung cancer. [file epih-46-e2024041-Supplementary-2.docx]

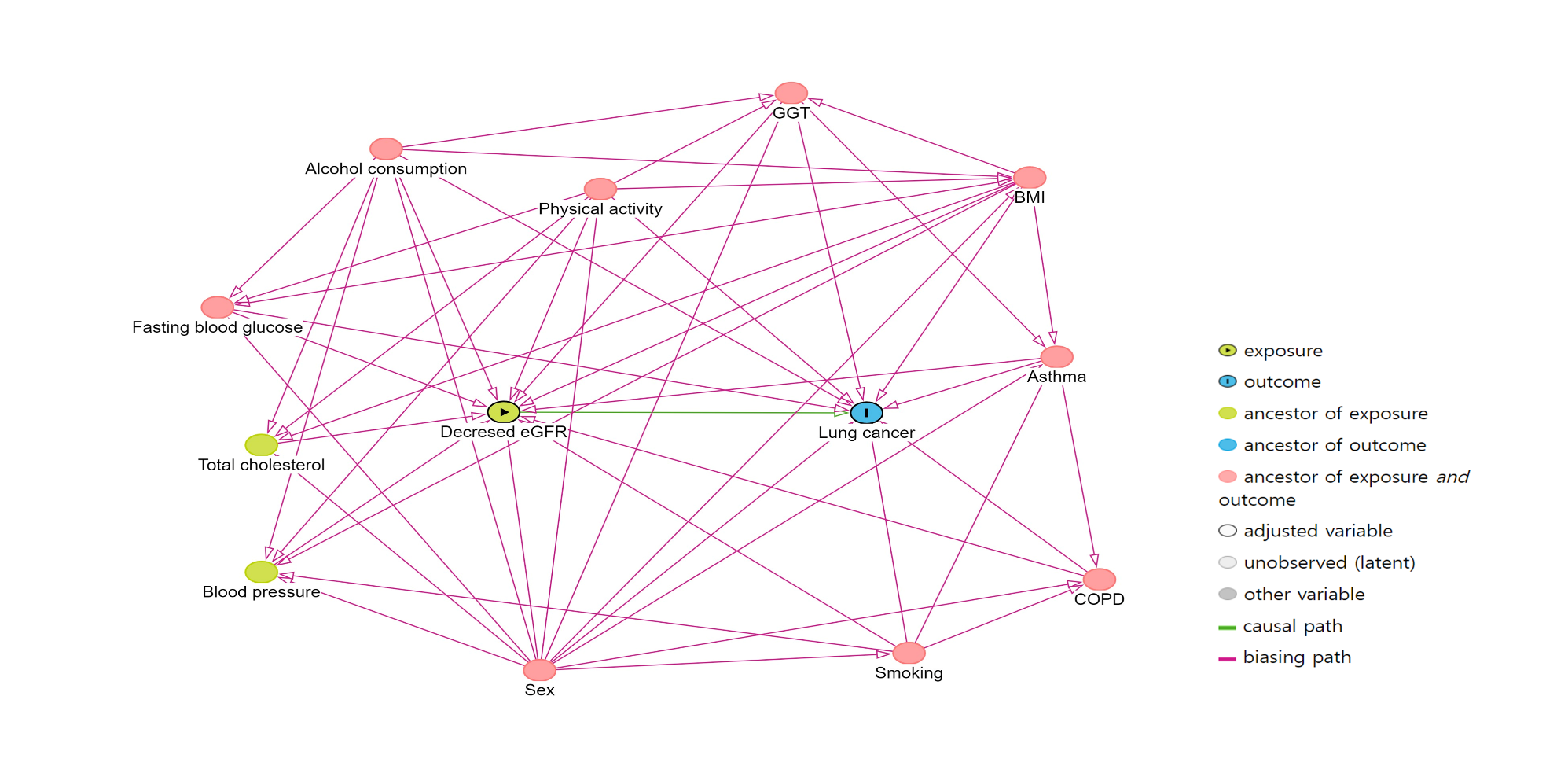


**Supplementary Material 2. Directed acyclic graph depicting the association between decreased eGFR, covariates and incident lung cancer.**

Abbreviations: eGFR, estimated glomerular filtration rate; BMI, body mass index; GGT, γ-glutamyltransferase; COPD, chronic obstructive pulmonary disease
