# Supplementary material for: Association of decreased estimated glomerular filtration rate with lung cancer risk in the Korean population
Source: Epidemiol Health. 2024 Mar 20;46:e2024041. doi: 10.4178/epih.e2024041 (PMC11369561; doi:10.4178/epih.e2024041)
Supplement: Supplementary Material 3. — Cumulative incidence of incident lung cancer by eGFR groups. [file epih-46-e2024041-Supplementary-3.docx]

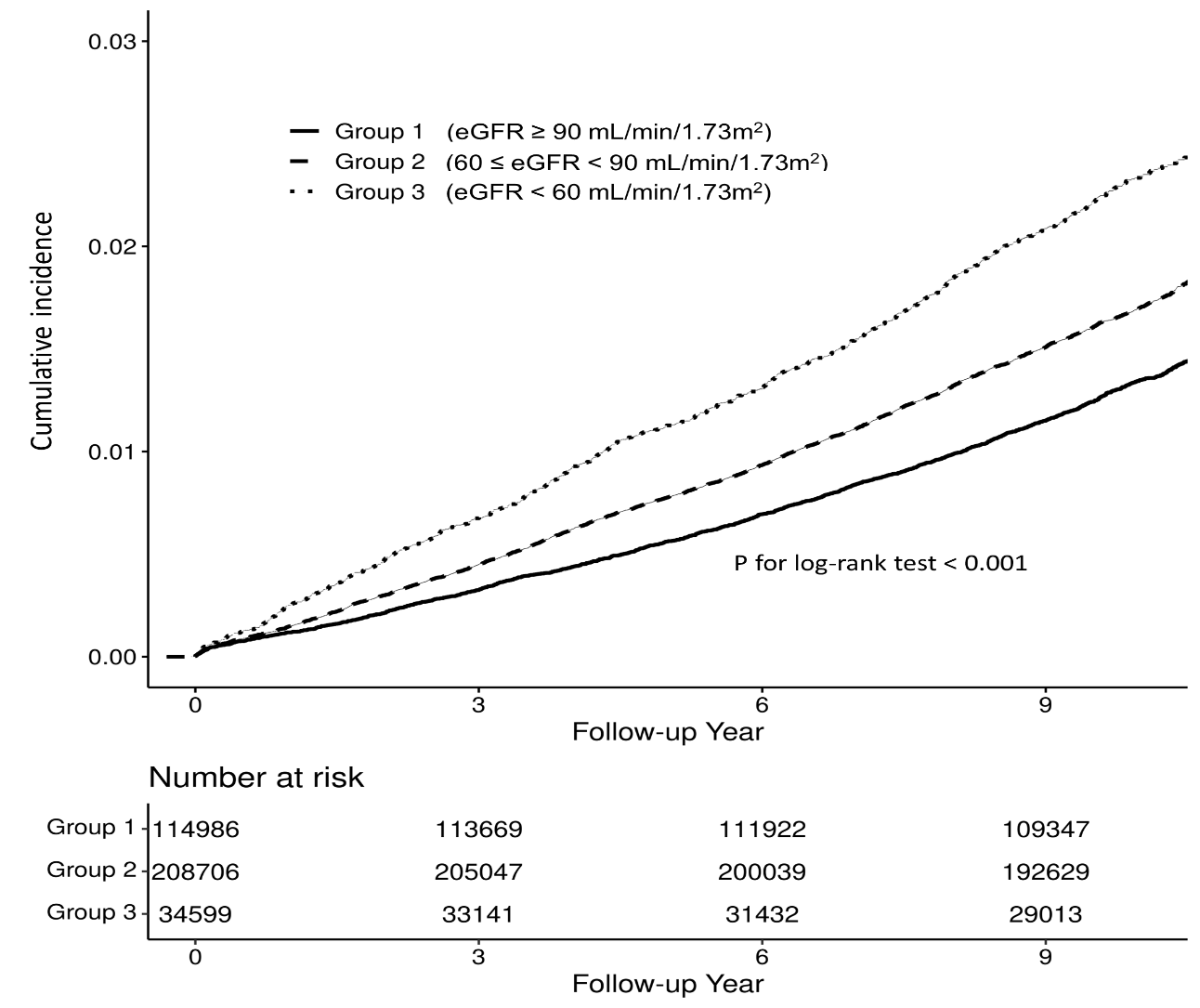


**Supplementary Material 3. Cumulative incidence of incident lung cancer by eGFR groups.**

eGFR, estimated glomerular filtration rate
