# Supplementary material for: Association of decreased estimated glomerular filtration rate with lung cancer risk in the Korean population
Source: Epidemiol Health. 2024 Mar 20;46:e2024041. doi: 10.4178/epih.e2024041 (PMC11369561; doi:10.4178/epih.e2024041)
Supplement: Supplementary Material 4. — Hazard ratios and 95% confidence intervals for the incidence of lung cancer according to the three groups of baseline eGFR categories after excluding the possibility of 1-year reverse causality [file epih-46-e2024041-Supplementary-4.docx]

**Supplementary Material 4. Hazard ratios and 95% confidence intervals for the incidence of lung cancer according to the three groups of baseline eGFR categories after excluding the possibility of 1-year reverse causality**

|  | Hazard ratios (95% confidence interval) | |
| --- | --- | --- |
|  | Unadjusted model | Multivariate-adjusted model |
| eGFR (mL/min/1.73m^2^) |  |  |
| Group 1 (≥90) | 1.00 (reference) | 1.00 (reference) |
| Group 2 (≥60–<90) | 1.28 (1.20–1.37) | 1.27 (1.19–1.36) |
| Group 3 (<60) | 1.72 (1.57–1.88) | 1.70 (1.54–1.87) |
| *P* for trend | <0.001 | <0.001 |
| Sex (female vs. male) |  | 0.59 (0.54–0.64) |
| BMI |  | 0.94 (0.93–0.95) |
| Fasting blood glucose |  | 1.00 (1.00–1.00) |
| GGT |  | 1.00 (1.00–1.00) |
| Smoking amount (pack-year) |  | 1.02 (1.02–1.02) |
| Smoking status |  |  |
| Never smoker |  | 1.00 (reference) |
| Former smoker |  | 0.77 (0.69–0.84) |
| Current smoker |  | 1.23 (1.12–1.35) |
| Alcohol consumption |  | 1.07 (0.99–1.16) |
| Physical activity |  | 1.02 (0.95–1.10) |
| COPD |  | 1.65 (1.56–1.75) |
| Asthma |  | 1.38 (1.29–1.48) |

The multivariate-adjusted model was adjusted for sex, BMI, fasting blood glucose, GGT, smoking amount (pack-years), smoking status, alcohol consumption, physical activity, COPD, and asthma.

Age is not included in the multivariate-adjusted model.

Abbreviations: eGFR, estimated glomerular filtration rate; BMI, body mass index; GGT, γ-glutamyltransferase; COPD, chronic obstructive pulmonary disease
