# Supplementary material for: Association of decreased estimated glomerular filtration rate with lung cancer risk in the Korean population
Source: Epidemiol Health. 2024 Mar 20;46:e2024041. doi: 10.4178/epih.e2024041 (PMC11369561; doi:10.4178/epih.e2024041)
Supplement: Supplementary Material 7. — Hazard ratios and 95% confidence intervals for the incidence of lung cancer to the three groups of baseline eGFR categories after stratifying by sex [file epih-46-e2024041-Supplementary-7.docx]

**Supplementary Material 7. Hazard ratios and 95% confidence intervals for the incidence of lung cancer to the three groups of baseline eGFR categories after stratifying by sex**

|  | Male (n = 194,063) | | Female (n = 164,230) | |
| --- | --- | --- | --- | --- |
|  | Hazard ratios (95% confidence interval) | | Hazard ratios (95% confidence interval) | |
|  | Unadjusted model | Multivariate-adjusted model | Unadjusted model | Multivariate-adjusted model |
| eGFR (mL/min/1.73m^2^) |  |  |  |  |
| Group 1 (≥90) | 1.00 (reference) | 1.00 (reference) | 1.00 (reference) | 1.00 (reference) |
| Group 2 (≥60–<90) | 1.26 (1.17–1.36) | 1.33 (1.23–1.43) | 1.16 (1.04–1.30) | 1.13 (1.00–1.26) |
| Group 3 (<60) | 1.74 (1.57–1.93) | 1.76 (1.58–1.96) | 1.73 (1.48–2.04) | 1.63 (1.38–1.92) |
| *P* for trend | <0.001 | <0.001 | <0.001 | <0.001 |
| BMI |  | 0.92 (0.91–0.93) |  | 0.98 (0.97–1.00) |
| Fasting blood glucose |  | 1.00 (1.00–1.00) |  | 1.00 (1.00–1.00) |
| GGT |  | 1.00 (1.00–1.00) |  | 1.00 (1.00–1.00) |
| Smoking amount (pack-year) |  | 1.02 (1.02–1.02) |  | 1.03 (1.01–1.04) |
| Smoking status |  |  |  |  |
| Never smoker |  | 1.00 (reference) |  | 1.00 (reference) |
| Former smoker |  | 0.74 (0.67–0.81) |  | 0.73 (0.38–1.40) |
| Current smoker |  | 1.14 (1.04–1.25) |  | 1.90 (1.36–2.64) |
| Alcohol consumption |  | 1.08 (1.01–1.16) |  | 0.87 (0.61–1.23) |
| Physical activity |  | 1.02 (0.94–1.11) |  | 1.01 (0.88–1.16) |
| COPD |  | 1.82 (1.71–1.95) |  | 1.32 (1.18–1.47) |
| Asthma |  | 1.44 (1.33–1.55) |  | 1.29 (1.15–1.44) |

The multivariate-adjusted model was adjusted for sex, BMI, fasting blood glucose, GGT, smoking amount (pack-years), smoking status, alcohol consumption, physical activity, COPD, and asthma.

Age is not included in the multivariate-adjusted model.

Abbreviations: eGFR, estimated glomerular filtration rate; BMI, body mass index; GGT, γ-glutamyltransferase; COPD, chronic obstructive pulmonary disease
